# Porous Silicon Optical Devices: Recent Advances in Biosensing Applications

**DOI:** 10.3390/s21041336

**Published:** 2021-02-13

**Authors:** Rosalba Moretta, Luca De Stefano, Monica Terracciano, Ilaria Rea

**Affiliations:** 1 National Research Council, Institute of Applied Sciences and Intelligent Systems, Unit of Naples, 80131 Naples, Italy; rosalba.moretta@na.isasi.cnr.it (R.M.); luca.destefano@na.isasi.cnr.it (L.D.S.); ilaria.rea@na.isasi.cnr.it (I.R.); 2 Department of Pharmacy, University of Naples Federico II, 80131 Naples, Italy

**Keywords:** optical biosensor, porous silicon, surface functionalization, immunosensor, aptasensor

## Abstract

This review summarizes the leading advancements in porous silicon (PSi) optical-biosensors, achieved over the past five years. The cost-effective fabrication process, the high internal surface area, the tunable pore size, and the photonic properties made the PSi an appealing transducing substrate for biosensing purposes, with applications in different research fields. Different optical PSi biosensors are reviewed and classified into four classes, based on the different biorecognition elements immobilized on the surface of the transducing material. The PL signal modulation and the effective refractive index changes of the porous matrix are the main optical transduction mechanisms discussed herein. The approaches that are commonly employed to chemically stabilize and functionalize the PSi surface are described.

## 1. Introduction

The term “biosensor” refers to an analytical and powerful tool made up of a bioreceptor (i.e., a biological recognition element) connected to a transducing substrate [1,2,3]. The bioreceptor is a biologically active material (i.e., enzyme, protein, antibody, oligonucleotide, and so on) responsible for the device selectivity; the transducer converts a specific biological recognition event (i.e., antibody-antigen interaction) into a measurable signal in real-time.

The first biosensor was made by Professor L.C. Clarck in 1956 for oxygen detection in the blood of patients undergoing surgery [4]. To date, incredible progress has been made both in technology and applications of biosensors, representing a very attractive research field, as demonstrated by the vast literature in the last 20 years.

It is expected that the biosensor market could reach U.S. Dollar (USD) 36.0 billion by 2027, due to the rising demand for such devices, with applications not only in biomedical diagnosis but also in environmental and food quality monitoring, industrial process control, agriculture, and others [5]. Moreover, the request for low-cost and user-friendly devices, with a fast-response time, is slowly replacing the currently available techniques for the identification of analytes, which are time-consuming, expensive, and require specialized laboratory equipment.

The design and the fabrication of the sensing system require the meticulous research of materials that have the desired transducer properties. In this context, nanostructured materials gained prominence in many applications because of their unique physicochemical characteristics over their bulk counterpart, such as high surface-to-volume ratio, small size, light absorption, optical sensitivity, and electrical and thermal conductivity [6,7]. Moreover, nanostructure-based biosensors show enhanced biosensing performances over conventional detection methods (i.e., higher sensitivity, fast response time, and low limit of detection (LoD)) [8,9,10]. Among the many available nanomaterials (e.g., quantum dots (QDs), metallic nanoparticles, carbon nanotube), porous silicon (PSi) has outstanding windows for applications in several research fields, from biosensing to drug delivery, thanks to its well-known optical and physical features [11,12,13].

Although it was accidentally discovered in 1956 by Uhlirs during an electrochemical experiment [14], this material received due attention only in 1990, when Canham discovered its intrinsic photoluminescence (PL) at room temperature [15]. Since the first experiments on PSi as a biosensor platform two decades ago, it is still an actual topic for research studies, as evidenced by the sustained number of scientific papers on PSi-based sensors published every year in peer-review journals [16].

PSi exhibits air-filled pores and a high surface area (up to 800 m^2^/g) [17]; these interesting characteristics, together with versatile surface chemical modification, tunable characteristic sizes, photoluminescence, biocompatibility, and biodegradability makes this material an appealing optical transducer [12,18,19,20,21,22]. Moreover, due to the high surface reactivity of PSi [23], several biomolecules can be easily immobilized within the porous matrix, by using well-established chemical approaches [13,24]. Since non-specific interactions reduce the selectivity of a biosensing platform, a blocking process of residual groups is generally performed as a final step of functionalization, by using agents like maleimide, bovine serum albumin, etc.

PSi is commonly obtained via electrochemical etching, a fabrication strategy that does not require expensive equipment, and allows a good reproducibility of the fabricated PSi substrates. This technique enables a fine control on the pore size and on the optical response of the material [25,26]. This tuning is generally not easily achievable in other porous materials, such as porous alumina [27,28] or porous titania [29,30].

Recently, PSi was also explored as a host matrix for the immobilization of several nanomaterials (i.e., QDs, graphene oxide, carbon dots) due to its high internal volume [31,32,33,34,35]. This feature made PSi more interesting than the other nanomaterials for biosensing applications. In fact, the possibility of combining different elements into the PSi matrix paved the way for the development of hybrid platforms that showed enhanced biosensing performances in terms of sensitivity, signal enhancement, signal stability, and dual-mode detection.

The optical response of PSi structures are strongly affected by their structural properties such as porosity (ratio of the fraction of voids in the layer to total volume), layer(s) thickness, and pore size and morphology [25]. Based on the International Union of Pure and Applied Chemistry (IUPAC) definition, three different PSi structures, with different pore sizes, could be distinguished—microporous Si (pore diameter d < 2 nm), mesoporous Si (pore diameter 2 < d < 50 nm), and macroporous Si (pore diameter d > 50 nm) [36]. Instead, the pore morphology is the least quantifiable aspect [25] and considers properties like shape (i.e., smooth, branched, facetted), orientation, and interconnection between pores. All these features are strongly influenced by the monocrystalline silicon and the anodizing regimes [25]. Macroporous and mesoporous Si are commonly employed as biosensing platforms to allow the attachment of biomolecules within their matrices. The capture of analytes can be monitored via reflectance, when dealing with macroporous or mesoporous structures (i.e., monolayer, Bragg mirror) [37,38], or via photoluminescence with mesoporous matrices (i.e., resonant microcavity, nanowires) [39,40]. Unfortunately, the intrinsic PL of PSi is usually unstable, since it strongly depends on the surrounding chemical environment that can influence its intensity [11]. For this reason, several works reported on the use of PSi substrates to embed emitting materials or molecules within pores, which sharpen the emission spectrum and amplify the signal [13,39,41].

Additionally, the versatile surface modification, optical tunability, low-cost fabrication, and label-free working conditions confer added value to this material. PSi is also compatible with microelectronics and MEMS fabrications systems [42,43,44]. Furthermore, the material’s biocompatibility allows the development of implantable biosensing devices that could be used for real-time detection of in vivo analytes [45].

PSi optical devices are widely used to detect different biomolecules (i.e., DNA, enzymes, cells, bacteria, antigen). Most PSi-based biosensors work in a label-free modality. The optical transduction principle relies on the change of the refractive index of the PSi layer, due to the substitution of air inside the pores with a target analyte. The change of the refractive index is detected as a wavelength shift of the corresponding reflectivity spectrum. Moreover, the intrinsic PL signal of PSi might be used as a further transduction mechanism for biosensing applications, by monitoring its variation upon a biomolecular recognition event. This review focuses on advances on the development of PSi-based optical biosensors achieved over the past five years. The fabrication process of PSi is briefly discussed, followed by an extensive description of the most common strategies used to stabilize and functionalize the material with biomolecules. Different examples of biosensor development, classified by biomolecules used as sensing elements, such as immunosensors, aptasensors, DNA hybridization, enzyme biosensors, and their application in the biomedical and environmental fields are examined [21,36,46]. Table 1 summarizes the detection of several biomolecules using PSi-optical devices.

## 2. PSi: From Fabrication to the Bioconjugation

**Figure 1:**
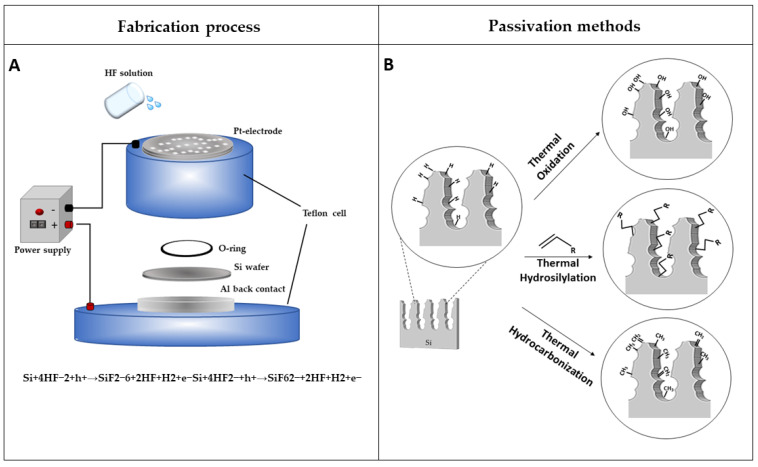
(**A**) Electrochemical etching setup; and (**B**) passivation strategies to stabilize PSi surfaces.

Although different strategies were developed to fabricate PSi structures, the anodic electrochemical etching of a crystalline silicon wafer in hydrofluoric acid (HF) water solution, remains the most commonly used. In 1956, Uhlirs first observed the formation of PSi during electrochemical procedures for polishing of silicon and germanium wafers [14]. This methodology is advantageous to other fabrication techniques (i.e., metal-assisted chemical etching [47,48], etc.), since it allows us to obtain different photonic structures with a high reproducibility [49]. The electrochemical etching is performed into an etching cell in which the silicon wafer acts as an anode and metals (e.g., platinum) act as a cathode. The current flow between the two electrodes leads to the dissolution of the crystalline structure, forming pores in the silicon wafer. The pore formation process is controlled by a complex mix of electronic and chemical factors [25]. A schematization of the electrochemical cell is shown in Figure 1A. Different porous silicon structures, with specific morphological and optical properties, can be engineered by changing the type of silicon wafer conductivity, doping level, concentration of hydrofluoric acid in electrolyte, applied voltage, current density, light intensity, and temperature [50,51]. Metal-assisted chemical etching (MACE) is another technique used for the fabrication of PSi. It is less common than the above-mentioned anodic etching. In a typical MACE process, a thin layer of noble metal (i.e., Au, Pt, etc.) sputtered on the silicon surface, catalyzes the etching of the material when placed in an oxidizing solution with hydrofluoric acid (HF). This technique generates several silicon nanostructures of tailored geometry, i.e., nanopores, porous silicon layers, nanowires, nanoneedles [52,53,54]

The freshly etched PSi is composed of hydride terminated groups (Si-H, Si-H_2_, and Si-H_3_), which make PSi nanostructure highly reactive and unstable. The aging effects, responsible for the uncontrolled growth of native oxide and the degradation of PSi matrix in alkaline or aqueous environments, strongly affect this material’s physicochemical and optoelectronic properties [55]. These effects might lead to zero-point drifts in the reflectivity spectrum and reduce, as a consequence, the sensitivity of the devices [25]. In this context, a proper surface chemical modification plays an important role to stop aging and to stabilize the PSi nanostructure (Figure 1B). Among the various developed treatments, the intentional growth of an oxide layer, under controlled conditions, represents one of the main approaches used to stabilize the hydrogen-terminated PSi surface. Despite the various available strategies for oxidizing the PSi matrix (i.e., ozone oxidation, electrochemical oxidation, and oxidation in aqueous solution), thermal oxidation is the most commonly used technique, by partially or fully replacing the reactive Si-H_x_ species into Si-O bonds [56]. This procedure is not only used to passivate the surface but also to convert a hydrophobic surface into a hydrophilic one. The rate of passivation is highly dependent on the temperature and on the duration of the treatment. Therefore, Shtenberg et al. demonstrated that the thermal oxidation of PSi nanostructures at 800 °C for 1 h, profoundly enhanced the sensitivity of the biosensor with respect to the process conducted at lower temperatures [57]. Moreover, the high temperatures allowed the conversion of the PSi structure into a silica skeleton. Due to its simplicity, the oxidation process represents the most used method to passivate PSi.

Other methodologies, involving the substitution of Si-H_x_ bonds into Si-C bonds, are proposed. The greater resistance of Si-C bonds to nucleophilic attack by water or hydroxide provides more excellent protection of the PSi matrix from physiological conditions and harsh basic environments. This process can be obtained by hydrosilylation, first demonstrated by Buriak et al. [23,31,32]. This approach is carried out by reacting liquid unsaturated compounds (i.e., terminal alkenes or alkynes) with Si-H_x_ species present on freshly etched PSi. This reactive mechanism can be promoted by heat, light, or chemicals (Lewis acid catalysis), and requires an inert atmosphere as well as deoxygenate/dried reagents.

The thermal carbonization of PSi represents another widely used approach to achieve highly stable Si-C bonds within the PSi nanostructures. Gaseous molecules as carbon sources (i.e., acetylene or ethylene) at high temperatures, can be easily adsorbed on the PSi, thus creating complete surface carbonization. The advantage of using this technique is the fast diffusion of gas molecules in small pores and the negligible steric hindrance with respect to liquid organic compounds [58]. Therefore, the temperature, used for the treatment, strongly influences the PSi surface termination—a hydrocarbon-terminated PSi is obtained at a temperature below 650 °C, generating a hydrophobic surface, due to some Si-H bonds, which are still present in the pores (thermally hydrocarbonized PSi, THCPSi); on the contrary, a completely carbonized PSi (thermally carbonized PSi, TCPSi) is obtained at a temperature above 800 °C, resulting in a hydrophilic surface due to the complete desorbing of hydrogen atoms from the PSi matrix [59].

The stabilization of the porous material, with the above-mentioned techniques, is a crucial factor for the development of stable platforms for biosensing purposes. The selectivity of the device is another essential element, achievable by using specific bioprobes that can be or is directly grown into the PSi matrix (in situ synthesis) or is synthetized ex situ, and is then, immobilized on the surface.

**Figure 2:**
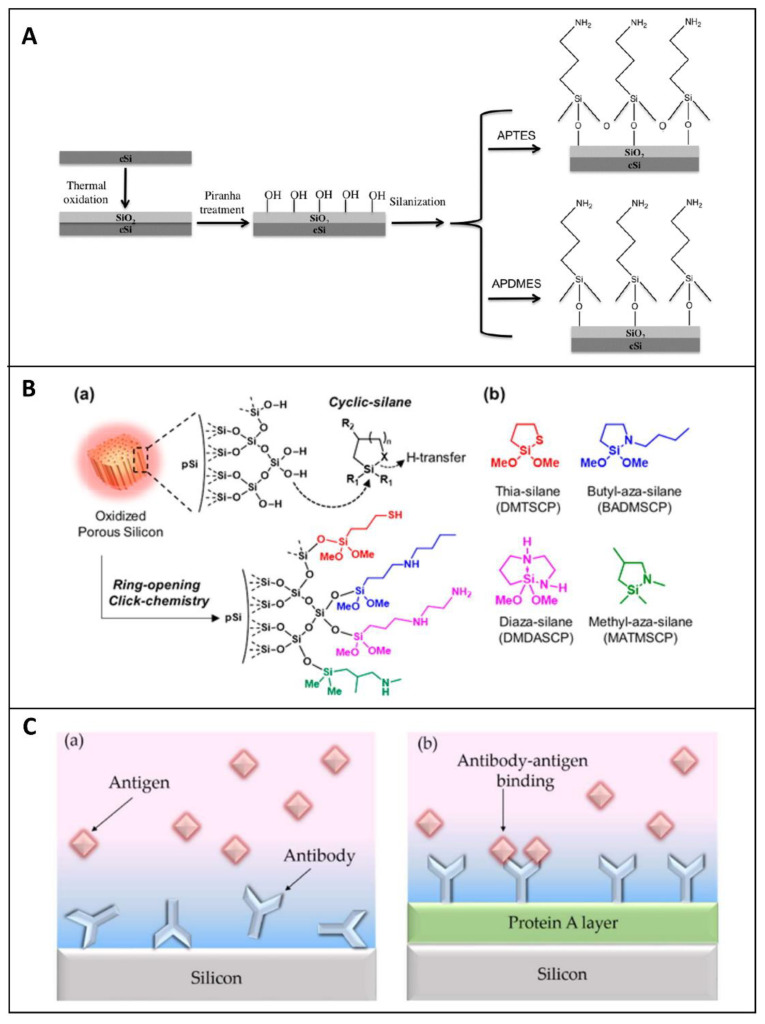
(**A**) Scheme of PSi functionalization through thermal oxidation and silanization with APTES or APDMES. Reprinted with permission from Reference [60]. (**B**) (a) Scheme of oxidized porous silicon modified through a ring-opening click reaction; (b) structure of reagents used for the study—thia-silane (DMTSCP, 2,2-dimethoxy-1-thia-2-silacyclopentane), butyl-aza-silane (BADMSCP, *N*-*n*-butyl-aza-2,2-dimethoxy-silacyclopentane), diaza-silane (DMDASCP, 2,2-dimethoxy-1,6-diaza-2-silacyclooctane), and methyl-aza-silane (MATMSCP, *N*-methyl-aza-2,2,4-trimethyl-silacyclopentane). R_1_ = OMe, Me. R_2_ = H, Me. Reprinted with permission from Reference [65] https://pubs.acs.org/doi/10.1021/jacs.6b08614 (accessed on 16 December 2020). Copyright (2016) American Chemical Society. (**C**) (a) Cartoons of randomly immobilized antibodies via physical adsorption and (b) properly orientated on the PSi matrix by using an intermediate layer of Protein A. Reprinted with permission from Reference [67] https://creativecommons.org/licenses/by/4.0/ (accessed on 16 December 2020).

After the PSi stabilization, different linkers can be used to graft biomolecules. The oxidized PSi can be readily modified via the silanization process (Figure 2A), based on the use of silane coupling agents (i.e., APTES, APDMES) characterized by different terminal motifs (i.e., NH_2_; SH, COOH; CHO) that act as anchorage sites for proteins, antibody, DNA and others [60]. Silane-based chemistry is one of the most exploited modifications of porous silica-based synthetic or natural materials since it offers an effortless way to attach a biological or chemical molecule to the porous surface [61,62,63]. Silanization requires the availability of hydroxyl groups on the PSi surface in order to hydrolyze the alkoxy groups of the alkyl silane molecule, thus forming Si-O-Si bonds [64]. Thiol or primary amino groups can be also used for grafting biomolecules. Their exposure on PSi surface can be obtained via a new silanization process, known as “ring-opening-click reaction”, proposed by Sailor et al., in which the heterocycles silanes, having Si-N or Si-S motifs in the ring, undergo a ring-opening reaction to modify the hydroxylated porous walls. The proposed surface chemistry, obtained in mild conditions and without the formation of by-products, does not interfere with the protein activity (Figure 2B) [65]. Although silanol chemistry is the most conventional approach for bioprobe immobilization, it is well known that Si-O-Si bonds are characterized by the lack of stability in aqueous and alkaline media, causing degradation of the coating surface, which is the main problem related to this chemistry.

Higher stability can be obtained through the Si-C bonds. The carboxyl acid group of undecylenic acid, as a result of the hydrosilylation process, are currently activated via 1-ethyl-3-[3-dimethylaminopropyl] carbodiimide hydrochloride (EDC) coupling agent for loading primary amine-containing biomolecules through direct reaction or via N-hydroxysulfosuccinimmide (NHS) [31]. Finally, the same surface chemical modification adopted for hydrosilylated-PSi can be applied for TCPSi and THCPSi. Sciacca et al. proposed a radical coupling reaction by using a radical initiator benzoyl peroxide and a dicarboxylic acid as a linker, generating a surface coated with carboxyl acid groups. This surface can be used to attach amino-terminal biomolecules via EDC/NHS chemistry [66].

The performances of a bio-device strongly depend on the right orientation of the biomolecules. This factor is particularly important for the immobilization of antibodies. Several research groups reported a valid strategy to optimize the biomolecules orientation on PSi matrix, by covering the surface with a layer of protein A. In such a design, the protein A interacts with the fragment crystallizable (FC) region of antibodies, leaving the antigen-binding fragment (Fab) region available toward the antigen (Figure 2C). This methodology allows proper anchoring of the antibody to the surface, maximizing its binding capability [67].

The stability and sensitivity of a biosensor are crucial when the target is in complex biological systems. In this context, the minimization of the unspecific adsorption of unwanted biomolecules is required. This aim can be achieved by using blocking agents to cap the available reactive sites (i.e., polyethylene glycol, maleimide, bovine serum albumin).

## 3. PSi Optical Transducer for Biosensors Development

**Figure 3:**
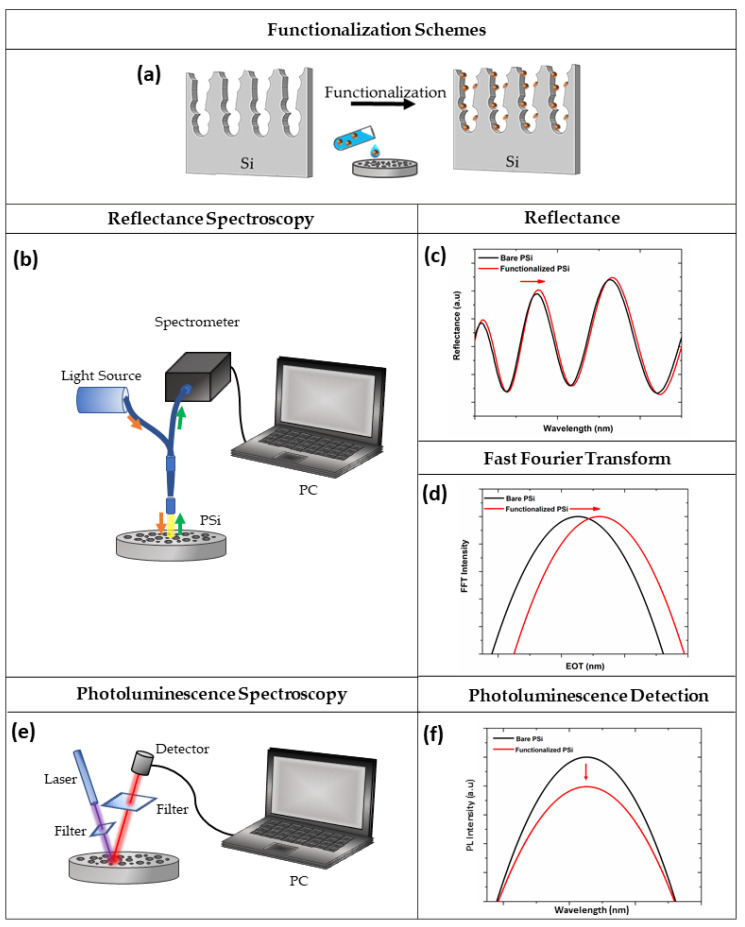
(**a**) Functionalization scheme of PSi; (**b**) optical setup for spectroscopy reflectometry, (**c**) reflectivity, and (**d**) FFT spectra before (black line) and after (red line) PSi functionalization; (**e**) optical setup for photoluminescence spectroscopy, and (**f**) corresponding spectra before (black line) and after (red line) the functionalization procedure.

The PSi structure is by far one of the most fascinating materials for the construction of a variety of biosensors, due to its optical, electrical, and chemical properties. To date, optical PSi biosensors are the most used label-free devices in biosensing, due to the low-cost production, high sensitivity, and real-time analysis. The label-free sensing mechanism of PSi optical structures is studied by interferometric reflectance spectroscopy (IRS), based on the monitoring of the refractive index (RI) changes on the photonic structure, induced by a specific bio-recognition event [68]. In the IRS set-up, an incident white light is reflected from the two typical interfaces of the PSi structure (air/PSi and PSi/Si bulk), generating a fringe pattern in the reflectance spectrum, known as Fabry-Pérot, which depends on the effective optical thickness (EOT = 2nL, where n is the average refractive index and L is the thickness of PSi layer). The Fabry-Pérot relationship is described by Equation (1):

(1) $$\begin{array}{c} m\lambda \;=2nL\; \end{array}$$

in which *m* is an integer and *λ* the wavelength of the incident light [69]. Therefore, the EOT is recorded by Fast Fourier Transform (FFT) of the reflectivity spectrum (Figure 3a–d). PSi is defined as a mixture of air and silicon in which the effective refractive index is dependent on the content of air inside the pores. When the air in the porous matrix is replaced with an analyte, an increase in the RI occurs, producing a shift of the reflectivity spectrum to longer wavelengths (red-shift). Moreover, a decrease in the average RI of the material causes a shift to shorter wavelengths (blue-shift); this phenomenon is generally ascribable to the oxidation/corrosion of the PSi skeleton [38,70].

Although all PSi devices are able to detect the presence of analytes within the pores, the optical response of the material can be finely tuned by changing the layers porosity, thickness, and number. The PSi monolayer is the simplest photonic structure used in biosensing, whose spectrum shows a periodic behaviour typical of Fabry-Pérot interferometer. However, more complex multi-layered structures with different porosities (i.e., Bragg mirror, microcavity, PSi rugate filter) can be fabricated, showing enhanced optical properties. For example, the reflectivity spectra of the Bragg mirror show a wide range of wavelengths having high reflectance (i.e., stopbands) whose maximum reflectance value is dependent on the number of layers in the structure. Moreover, to detect the infiltration of analytes into the pores, the spectral position of one edge of the stopband is monitored [71,72]. The typical narrow spectral feature of a microcavity makes this photonic structure easy to control upon molecular infiltration, with respect to the Bragg mirror. Moreover, as previously reported, PSi exhibits an intrinsic PL signal at room temperature. Therefore, the modulation of this signal, caused by a biological recognition event, might be employed for biosensing purposes. A scheme of the optical setup for PL measurements is shown in Figure 3e,f.

The main drawback of label-free, PSi-based biosensors is a low sensitivity, in the micromolar range, due to the slow and limited mass-diffusion inside the matrix. This limit is overcome through the fabrication of more complex and multi-layered optical structures (i.e., Bragg mirror, rugate filters, microcavities, ring resonators). An example is provided by the open-ended PSi microcavity membrane that allows a fast response time and a more favourable interaction between the analyte and the inner surface [73,74]. Moreover, the sensitivity of PSi-based biosensors can be greatly improved by optimizing the experimental platform engineering [75,76], data processing methodologies [77,78], as well as signal amplification mechanisms [79]. Moreover, the PSi matrix can be integrated with inorganic materials (i.e., quantum dots, graphene oxide, titanium dioxide) allowing the realization of hybrid devices with advanced properties, and improving the biosensing performances (i.e., sensitivity, stability, the limit of detection). In the following sections, the realization of PSi-based devices are reported, highlighting the enormous versatility of PSi as a transducer surface for the development of different types of biosensors.

### 3.1. Porous Silicon Immunosensors

The antibodies represent the most common probes used in biosensing systems. Specific antibodies or their fragments (fragment binding antigen-Fab) can be used as biosensor molecular recognition elements against precise antigens. The development of hybridization and cloning techniques allowed the production of a variety of antibodies, paving the way for the development of different immunosensors. The antibodies are widely used in PSi-based devices for the detection of several analytes.

The development of a highly sensitive PSi immunosensor needs a careful study on several working parameters, such as the antibody orientation onto PSi surface, the analyte diffusion into the matrix, as well as the choice of the photonic structure [67,80].

**Figure 4:**
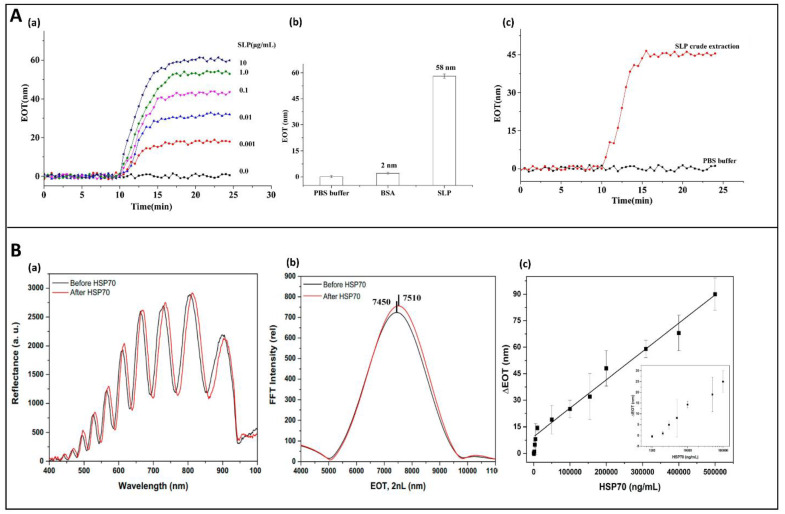
(**A**) (a) EOT signals of anti-SLP/ProteinA/TiO_2_-PSi-based immunosensor after incubation in different SLP concentrations (from bottom to up). (b) Specificity test of TiO_2_-PSi-microfluidic biosensor measured via EOT response to BSA and SLP. (c) SLP detection in a dilute crude extract. Reprinted with permission from Reference [80]; Copyright (2020) Elsevier B.V. (**B**) (a) Reflectivity spectra and (b) corresponding Fourier Transforms (FFT) of anti-HSP70-PSi device before (black line) and after (red line) HSP70 detection at concentration of 200,000 ng/mL. (c) Relationship between HSP70 concentrations and EOT shifts (inset semi-log plot). Reprinted with permission from Reference [83]; Copyright (2020) Elsevier Ltd.

Zhuo et al. reported the development of a hybrid TiO_2_–PSi-based immunosensor for the rapid detection of the S-layer protein (SLP), a surface protein available on the cell walls of several bacteria [81]. The device was obtained by spinning TiO_2_ solution onto the PSi and putting it in a furnace at 500 °C for 1 h [82]. The as-obtained hybrid platform was integrated into a microfluidic system, in which, first, protein A and then, anti-SLP were pumped through the cell. Finally, SLP was injected into the system. The protein A, adsorbed on the surface, was used as a spacer to reduce the steric hindrance and improve the antigen–antibody interaction. The following system demonstrated an increased sensitivity thanks to the high refractive index of the material and the dynamic sampling system, reaching an LoD of 0.70 ± 0.37 pM [80]. Some results are reported in Figure 4A.

The antibodies were also exploited as bioprobes for the direct detection of whole-cell organisms such as bacteria or viruses. A recent work of Tang et al. reported the *Escherichia coli* detection by using a nanopore array as a sensing platform, upon functionalization with *E. coli* antibody and integration into a microfluidic system. The oxidized PSi was activated by NaOH, followed by HCl treatment and washings in deionized water. After silanization with Amino-propyl-triethoxy-silane (APTES) and modification with glutaraldehyde (GA), *E. coli* antibodies were attached to the surface. The biosensing principle was based on the direct capture of *E. coli* on the immunosensor, reducing the pore accessibility, which was measured through indirect Fourier Transformed Reflectometric Interference Spectroscopy (FT-RIS). A bacterial density, ranging from 10^3^ to 10^7^ colony-forming unit (CFU) mL^−1^ was linearly correlated to a decrease in EOT shift [84].

Recent studies focused on the heat shock protein 70 (HSP70), a molecular chaperone found in eukaryotic cells, with anti-apoptotic role, whose high expression was correlated to several types of cancers [85]. Thus, the monitoring of HSP70 levels could be useful for the early detection of this pathological condition. Although several electrochemical and optical biosensors were already developed for HSP70 detection, the high costs and the complex procedures for the preparation of the platforms made necessary the development of an alternative device [86,87,88]. In this context, Manyia et al. [83] reported a proof-of-concept study for the fabrication of a low-cost and label-free optical immunosensor for HSP70. After PSiO_2_ surface silanization and subsequent modification with GA, an anti-HSP70 antibody was grafted within the pores. The detection of HSP70 reveals an LoD of 1290 ± 160 ng/mL, showing a sensitive detection in the range of 3000–500,000 ng/mL (Figure 4B). Moreover, the sensitivity of this platform could be improved after its integration into a microfluidic system.

The intrinsic PSi PL was employed for the development of biosensors, by monitoring the quenching or the enhancement of the signal due to a biomolecular interaction. In this context, a PL-immunosensor was developed by Myndrul et al. for the rapid detection of a fungal mycotoxin, Ochratoxin A (OTA), a major source of food and beverages contamination [89,90]. The detection of OTA was conducted through a specific antibody. In particular, the PSi surface, obtained by using the MACE procedure [91], was modified with APTES, GA, and Protein A. Finally, an anti-OTA was covalently immobilized to the support. The specific interaction between OTA and immunosensor caused quenching of the PL signal, with a response time in the range of 500–700 s and an LoD of 4.4 pg mL^-1^ [92]. On a similar principle, Irrera et al. demonstrated the development of a label-free optical Si nanowires (NWs) biosensor to detect the C-reactive protein (CRP). The high value of this protein in the blood is a synonym of a phlogistic condition, often an index of cardiovascular diseases. The high biotin-streptavidin affinity was exploited to immobilize a biotinylated anti-CRP to the Si NWs modified with streptavidin. The detection of CRP concentration was evaluated via PL quenching caused by the CRP-anti-CRP-biosensor interaction. The high selectivity of the platform towards the CRP allowed reaching an LoD of 1.6 fM [93].

Hybrid platforms, made of PSi substrate and gold (Au), are proposed as an alternative methodology to detect mycotoxins in food. Myndrul et al. reported the development of a PSi/Au immunosensor for the detection of Aflatoxin B1 (AFB1), one of the most common aflatoxin with toxic effects on humans and animals. The platform was obtained by covering PSi substrate with a thin layer of gold, via chemical (PSi/Au_chem_) or electrochemical (PSi/Au_El_) deposition. To immobilize an anti-AFB1, PSi/Au substrates were functionalized with 11-mercaptoundecanoic acid (MUA), followed by the activation of the carboxyl acid groups of MUA via EDC/NHS chemistry. At this step, the samples were immersed into a solution of protein A in sodium acetate buffer and then into an anti-AFB1 solution. Variation in the PL intensity signal was used to detect the presence of AFB1. In particular, when analyte concentration increased, a decrease of the PL signal was detected. The biosensor showed an LoD value of about 2.5 ± 0.5 pg/mL and a high sensitivity of PSi/Au_El_ towards AFB1 in the range of 0.01–10 ng/mL [94].

Innovative immunosensors were obtained by confining QDs into a PSi structure, in order to evaluate the fluorescence enhancement [35,95,96]. In particular, Li et al. proposed a biosensor for the detection of *Echinococcus granulosus*, where the hydatid antigen (Egp38) was immobilized into a Bragg mirror surface. The preparation of the device consisted of the PSi oxidation, through sample immersion in 30% hydrogen peroxide, silanization with APTES, and immobilization of Egp38 antigen through GA. QDs were covalently attached to the hydatid antibody (rabbit anti-p38). The biological reaction occurring between the antigen and QDs-antibody could be detected through a fluorescence signal whose response linearly depended on the antigen concentration, with an LoD of 300 fg mL^−1^ [35].

### 3.2. Enzyme-Based Porous Silicon Biosensors

An enzyme is commonly defined as a “biological catalyst”, a protein able to decrease activation energy of specific chemical reaction. The enzymatic reactions show a very high specificity because of the stereospecific interaction between the enzyme active site and the corresponding substrate. The enzymatic activity can be evaluated by measuring either the substrate depletion or the product formation rate. Furthermore, an enzyme can also be used as biorecognition elements.

The enzyme-based biosensors provide many advantages, such as high sensitivity, specificity, and cost-effectiveness, giving the possibility to develop a point-of-care diagnostic (POC) device with applications in many research fields, including food safety control and environmental management, and clinical diagnosis.

Nowadays, heavy metals pollution is one of the most severe environmental problems, due to their large use in agriculture and the industrial field, with dramatic consequences for the human health. Since the most commonly used techniques for heavy metals monitoring are time-consuming and require laborious procedures [107,108], Segal et al. developed an alternative label-free methodology based on oxidized-PSi nanostructures functionalized with horseradish peroxidase (HRP). The reported device was obtained through the oxidation of PSi, followed by the amino-modification of the surface via APTES and diisopropylethylamine (DIEA), and finally, through activation with bis(N-succinimidyl)carbonate (DSC) crosslinker, which is useful to graft the enzyme through the lysine groups. By using this device, the catalytic activity of the enzyme can be monitored by RIFTS in real-time. The active site of the HRP showed a conformation change after heavy metals binding, thus inhibiting the enzymatic activity. The reported biosensor showed a sensitivity versus Ag^+^, Pb^2+^, and Cu^2+^, measuring an LoD of 60–120 ppb. Moreover, the specific detection of Cu^2+^ ions was demonstrated by immobilizing the laccase on a PSi platform, showing comparable results with respect to those obtained by inductively-coupled plasma atomic emission spectroscopy (ICP-AES) [97].

Enzymatic biosensors might be useful not only for the identification of pollutants in the environment but also for the detection of diseases such as cancer or bacterial infections.

**Figure 5:**
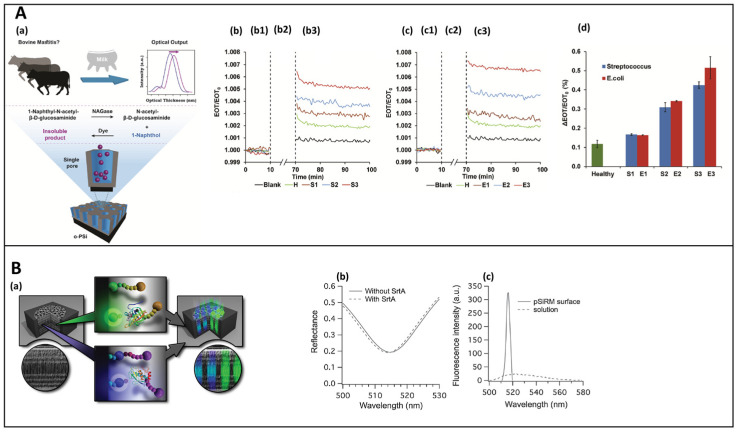
(**A**) (a) Schematic representation of o-PSi biosensor for the NGAse activity detection. The NGAse present in the milk hydrolyzes the NGAse substrate into N-acetyl- β-D-glucosaminide and 1-naphthol. The reaction between 1-Naphthol and Hexazonium Pararosaniline forms an insoluble product that precipitates into the PSi nanostructures causing a change in the reflectivity spectra; (b) reflective-based response of NGAse catalytic activity in milk samples positive to *S. dysgalactiae* and (c) *E. coli*. The lines (b1, c1) are the baseline, the reaction solution and the dye are represented by lines (b2, c2) while the buffer wash is represented by lines (b3, c3); (d) averaged optical response obtained by subtracting the blank relative EOT for the corresponding milk samples. Reprinted with permission from Reference [98]; Copyright (2020) American Chemical Society. (**B**) (a) Scheme of a FRET-based PSi biosensor for the detection of SrtA and MMP. The FRET peptide substates are immobilized into a PSi microcavity (central panels). The enzymatic cleavage causes a fluorescence enhancement (right panels); (b)reflectance spectra of PSiRM before and after incubation with SrtA; (c) fluorescence emission of SrtA substrate in solution (dashed line) and immobilized on the PSi surface after SrtA incubation. Reprinted with permission from Reference [39] https://creativecommons.org/licenses/by/4.0/ (accessed on 16 December 2020).

In a recent study, the lysosomal activity of N-acetyl-β-D-glucosaminidase (NGAase) in real milk sample was monitored for the identification of bovine mastitis, an inflammatory process often caused by *E. coli* and *Streptococcus dysgalactiae.* The platform was obtained through thermal oxidation of Psi, followed by amino-modification with APTES and DIEA, to allow cross-linking with DSC. Finally, a solution of gelatin was applied on the support. The as-obtained gelatin-functionalized PSi matrix (o-PSi) was used to monitor the biochemical activity of NGAase in the milk, in presence of the NGAase substrate via the RIFTS technique. The reaction products accumulate inside the porous structure causing a change in the refractive index of the material and, as a consequence, a shift of the fringes in the optical spectrum (Figure 5A). Obviously, the optical response was correlated to the concentration of NGAase in the milk. The following device, with an LoD of 0.51 µM/min, is a simple and portable platform with promising applications in the diagnostic field [98].

Several works remarked the fluorescent molecules embedding into PSi microcavity biosensors to enhance the fluorescence signal of the fluorophores, obtaining an improved sensitivity and LoD of the device [109]. This approach was widely presented by Voelcker et al. who reported the development of a porous silicon resonant microcavity (PSiRM), with the aim to detect Sortase A (SrtA), a membrane-anchored transpeptidase involved in the virulence process of *Staphylococcus aureus*. A fluorogenic SrtA peptide substrate was modified with Dnp and FITC, the former used as a quencher while the latter as a fluorescent dye. The substrate was covalently immobilized on the PSi structure, previously stabilized through thermal hydrosilylation. The enzymatic activity of SrtA caused the cleavage of the substrate and the removal of the quencher, generating an FITC fluorescence with a 13-fold enhancement with respect to the non-cleaved substrate. While the LoD achieved with the reflectance analyses was 4.6 × 10^−8^ M, the fluorescence detection guaranteed an LoD of 8.0 × 10^−14^ M. This result could be explained through the Purcell effect—the microcavity confines the light and enhances the emission of the fluorophores inside the pores [110,111]. Moreover, the combination in a single array of two different fluorogenic substates, one specific for SrtA and the latter for the MMP−1, allowed the development of a multiplexing detection of biomarkers, as shown in Figure 5B [39,112].

The same group reported the detection of the enzyme L-lactate dehydrogenase (LDH), normally found in human tissues. High LDH levels in the blood are indicative of pathological conditions. Since the upregulation of LDH is associated with leukemia, melanoma, pulmonary cancer, and chronic wounds, a rapid assessment of LDH levels in the blood is fundamental for the early diagnosis of these diseases. To this aim, the authors reported the use of resazurin-modified PSiMC acting as a luminescence-enhancing optical biosensing platform for LDH monitoring. LDH is an enzyme that catalyzes the conversion of L-lactate to pyruvate and the reduction of NAD+ into NADH. This reaction was coupled to the reduction of non-fluorescent resazurin into fluorescent-resorufin [113]. For the platform development, PSiMC was passivated via thermal hydrocarbonization and thermal hydrosilylation, followed by the formation of acid chloride by using thionyl chloride and acylation of a non-fluorescent resazurin. The passivation procedure strongly improves the surface stability, preventing both the oxidation of PSiMC and resazurin, prior to detection. The LDH detection through resazurin-PSiMC was monitored as an increase in fluorescence intensity of 10 and 5-fold higher, with respect to that of single-layer and detuned microcavity, respectively. The reported platform showed an LoD of 0.08 U/mL and a detection range between 0.16 and 6.5 U/mL, which covered the concentration range of the enzyme in healthy and damaged cells [99].

Ghosh et al. explored the PL tunability of Si NWs, after their surface modification by glucose (GL) and by its conjugated enzyme, glucose oxidase (GO_x_). The surface modification of Si NWs was obtained by dipping the samples in GL/GO_x_ solution for 1 h at 60 °C. A different PL effect was measured based on the different glucose concentrations. In particular, the H_2_O_2_ production in the GL/GO solution caused a PL quenching of Si NWs at lower GL concentrations; on the contrary, an enhancement of PL was observed at higher GL concentration—the different behaviour could be correlated to the removal of Si-H bonds on Si NWs surface and to the attachment of different carbon-based functional groups. Therefore, the proposed biosensor showed a detection range from 0.5 to 1.0 mM and an LoD of 1.06 µM [40].

### 3.3. Oligonucleotides- and Analogues-Based Biosensors

Oligonucleotide based biosensing technologies represent a field under intense investigation due to their broad applications in biomedicine and environmental monitoring.

An oligonucleotide-based biosensor shows a short functional nucleic acid, generally identified in a single-stranded DNA (ssDNA) or RNA, which is immobilized on the surface of the transducer material and specifically interacts with a complementary DNA or RNA sequence, through the base pairing. The same hybridization mechanism is outperformed by analogues of oligonucleotides (i.e., peptide nucleic acid, PNA), that have had a great impact in biosensing because of their enhanced properties, due to traditional oligonucleotide probes, opening the way for the development of highly specific platforms for the early detection of genetic diseases. Finally, aptamers are single-strands of DNA or RNA, commonly known to have a high affinity towards biological (i.e., whole cells, protein) or chemical compounds (i.e., metal ions). The high binding affinity towards the target makes the aptamers a valid alternative to antibodies. In all reported cases, the specific hybridization between the bioprobe and the respective target is detectable as an optical measurable signal.

#### 3.3.1. DNA and Peptide Nucleic Acid-PSi Biosensors

In recent years, there has been a substantial development in DNA-based biosensors due to the stability and sensitivity that distinguish these kinds of probes. In a DNA-PSi based biosensor, the interaction between the probe and the target is monitored as a red-shift in the reflectivity spectra, due to an increase in the average refractive index of the material [76,114]. However, many published articles report a shift of the spectrum at lower wavelengths when DNA/DNA hybridization occurs—this effect could be related to the corrosion of PSi structure, caused by the accumulation on the PSi surface of the DNA backbone negative charges [115,116]. Moreover, the corrosion process was controlled by Weiss et al. In particular, they obtained a greater stable surface, also in harsh environmental conditions, passivating the PSi surface through thermal carbonization. As a result, a 16-mer ss-DNA, covalently attached to TCPSi, was efficiently hybridized to the complementary DNA, minimizing the unspecific interactions [58].

**Figure 6:**
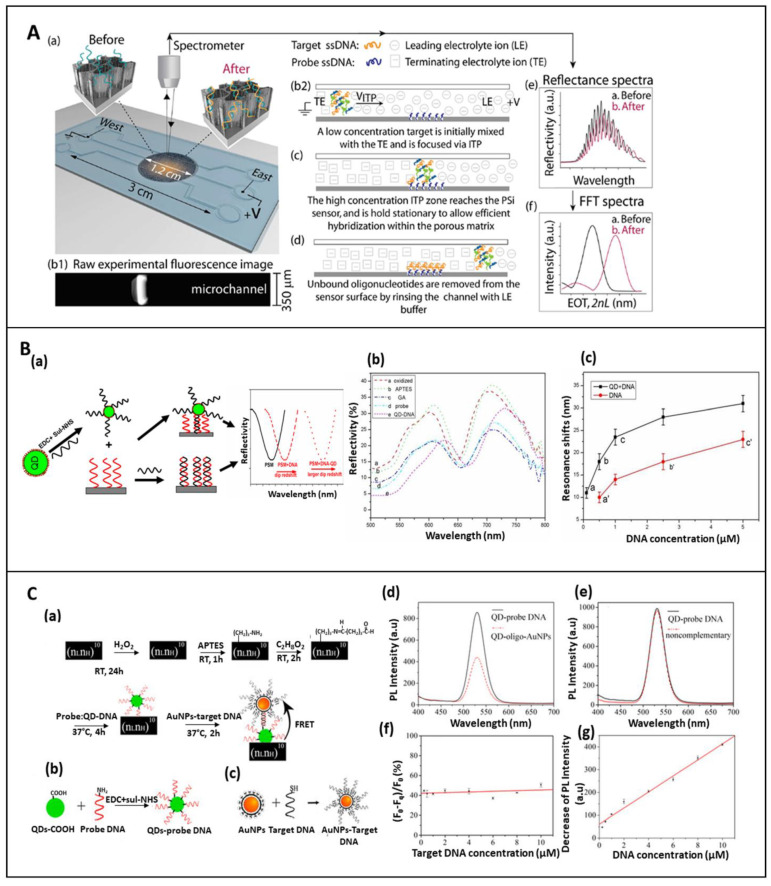
(**A**) (a) Scheme of the integrated PSiO_2_ interferometric biosensor combined with the ITP technique into a microfluidic system; (b–d) scheme of the concentration process of target DNA (ssDNA) under ITP, followed by binding with immobilized DNA probe; (e,f) reflectivity spectra and corresponding FFT before and after DNA hybridization. Reprinted with permission from reference [100]; Copyright (2015) Wiley-VCH. (**B**) (a) Schematic representation of the sensing principle—the hybridization between target QDs-DNA and the complementary sequence, immobilized into PSi microcavity, causes a huge red-shift of the reflectivity spectra, increasing the response signal; (b) reflectance spectra after each functionalization steps; (c) relationship between the of QDs-DNA and control DNA at different concentrations and reflectance spectra. Reprinted with permission from Reference [101] https://creativecommons.org/licenses/by/4.0/ (accessed on 16 December 2020). (**C**) (a) Functionalization steps and sensing mechanism of QDs-AuNPs FRET biosensor for DNA detection; (b) DNA conjugation to QDs, and (c) DNA conjugated to AuNPs; (d) PL emission of QDs embedded in PSi matrix before and after hybridization with AuNPs-DNA; (e) control experiment performed by using AuNPs-conjugated with non-complementary DNA; (f) quenching efficiency Q = 1 − F_q_/F_0_ with several DNA target concentrations; and (g) linear relationship between decrease of PL intensity and different AuNPs-target DNA. Reprinted with permission from Reference [103]. https://creativecommons.org/licenses/by/4.0/ (accessed on 24 January 2021).

The low LoD of PSi biosensors in the micromolar range, due to the limited mass transport of the analytes into pores, was exceeded by incorporating the biosensing architecture into a microfluidic system [75,76]. Moreover, a three-order magnitude enhancement in the DNA-PSi-based biosensor was measured by implementing the electrokinetic isotachophoresis (ITP) strategy, developed by Segal et al., into a microfluidic platform. By using an applied electric field, the charged analytes were separated on the basis of their ionic mobility, leading to the sample preconcentration, and as consequence, a great improvement in terms of the device sensitivity was achieved. A schematic representation of the PSi device integrated with ITP is shown in Figure 6A. The device was integrated into a microfluidic system after the PSi oxidation and its silanization with APTES. All other surface modifications were carried out by pumping the solutions into the microchannel. In particular, the NH_2_- PSi was incubated with a solution of sulfo-SMCC crosslinker, used to bound the thiol-modified ss-DNA. This technique allowed to improve the sensitivity of Fabry-Pérot biosensor of 1000-fold, measuring an LoD of 1 × 10^−9^ M [100].

The design of sophisticated photonic structures (i.e., ring resonator, Bragg mirror, waveguides) represents a further strategy to enhance the detection of low concentrated analytes. This issue might be pursued by confining the incident light and increasing the interaction between light and biomolecules. A recent example was described by Zhang et al. They reported a double Bragg mirror for detecting ammonia-oxidizing bacteria (AOB), microorganisms converting organic nitrogen into nitrite and nitrate. This PSi structure, obtained by electrochemical etching at room temperature, showed a deeper resonance peak and a broader reflectivity stop band in the reflectance spectrum, guaranteeing high sensitivity and specificity of the structure for biosensing applications. A DNA sequence, specific for AOB bacteria detection, was immobilized onto oxidized transducer material through APTES and GA and used to monitor the hybridization with either complete and partial complementary DNA sequences. The reporting LoDs were 27.1 nM and 35 nM, respectively, providing the feasibility for AOB detection in real environment [71].

The in situ ssDNA probe synthesis represents an alternative technique employed to enhance the density of capture sites in PSi photonic biosensors. Using this approach, Weiss et al. demonstrated a 5–7-fold enhanced sensitivity and a 3-fold reduction in response time in silicon microring resonators and photonic crystals, with respect to the traditional conjugation strategy. The in situ synthesis, performed by growing uncharged DNA monomers, base-by-base, reduced the steric hindrance and charge repulsion during the ssDNA immobilization [117]. In this context, the pore size of the PSi matrix represents a parameter that should be evaluated. Terracciano et al. reported the impact of pore size on the functionalization yield of PSi transducer, through an in situ synthesis approach. After the in situ synthesis of a 19-mer DNA sequence onto mesoporous PSiO_2_, the authors measured a medium-yield process. This effect was related to the pore size (~20 nm); a macroporous PSi with pore diameters larger than 50 nm resulted in maximizing the yield of in situ synthesis and the sensing performance of the device [38].

The sensing performance of the PSi device could be enhanced, in terms of sensitivity and signal stability, using the PSi platform as a host matrix for the incorporation of several compounds or nanomaterials, such as quantum dots (QDs), metals, polymers or fluorescent molecules. In this context, Lv et al. proposed a biosensor in which QDs were embedded into PSi structure. The DNA probe, immobilized onto oxidized PSi microcavity through GA, specifically hybridized with the target DNA, coupled to CdSe/ZnS QDs, improving the sensitivity of PSi device 5 times, compared to the unlabelled-DNA hybridization [101]. This effect could be related to the higher refractive index of QDs. A schematization of the sensing principle and some of the results are shown in Figure 6B.

Wei et al. proposed a novel, fast, and low-cost method to detect DNA/DNA interaction based on the digital imaging of PSi/QDs surface fluorescence. The authors reported the covalent grafting of a ssDNA into a Bragg mirror substrate. To modify the PSi surface, the sample was soaked in 30% H_2_O_2_, followed by silanization with APTES, modification with GA, and finally DNA attachment. Moreover, the carboxyl acid groups of CdSe/ZnS QDs were used to immobilize the ssDNA-target via covalent chemistry. To detect hybridization, the PSi surface was imaged by digital microscopy and, then, the images were analyzed by an image processing software. Data revealed an LoD of 88 pM, with a sensitivity slightly lower than that of conventional fluorescence analyses, but higher than the one obtained from reflectance spectroscopy. Therefore, the proposed approach is fast, low-cost, and convenient for detecting biological interactions [102].

An alternative approach to detecting an 16S rRNA, specific to Actinobacteria, was proposed by Zhang et al. These classes of bacteria are involved in the turnover of the organic matter and xenobiotics and they are significantly present in several habitats. Several methodologies were already proposed to detect DNA or RNA in food, environment, and other matrices. Moreover, although most of the proposed techniques are accurate, they are generally time-consuming and expensive [118,119]. The authors proposed a PSi biosensor based on the FRET mechanism between QDs and gold nanoparticles (AuNPs) in which the former act as an emission donor and the latter as a fluorescence quencher [120]. For the device realization, the PSi DBR was oxidized in H_2_O_2_ solution, silanized with APTES, modified with GA and, finally, QDs-DNA were bound onto the PSi substrate. Then, AuNPs were conjugated to the DNA target via thiol chemistry. To perform the hybridization experiment, different concentrations of AuNPs-DNA were added to the modified-PSi device, resulting in a quenching of the fluorescence intensity, which revealed to be proportional to the concentration of c-DNA in the range from 0.25 to 10 µM. Moreover, an LoD of 328.7 nM was achieved (Figure 6C), obtaining a simple, flexible, highly sensitive, and responsive platform [103].

Recently, peptide nucleic acid (PNA), a synthetic nucleic acids analogue, was employed as a bioprobe in oligonucleotides targeting. The uncharged PNA backbone, characterized by N-(2-aminoethyl)glycine motifs linked by peptide bonds, provides a stronger interaction between PNA/DNA strands in comparison to DNA/DNA duplex [121,122]. Consequently, a DNA/PNA base mismatch would be more destabilizing with respect to DNA/DNA complex. Other properties, such as the capability to hybridize in low-salt concentration as well as the chemical and thermal stability make the PNA a powerful probe to discriminate single-point mutations, allowing the development of biosensors for early diagnosis of genetic diseases.

One of the examples in the use of PNA as bioprobe in a PSi platform was reported by Weiss et al. They tried to reduce the corrosion process of the PSi matrix, normally induced by DNA/DNA hybridization, by replacing DNA molecules with a charge-neutral PNA probe and shelled the negative charge on the DNA backbone by the introduction of Mg^2+^ ions in the hybridization medium [115]. The same group later reported the design of a ring resonator, patterned with a PSi slab waveguide. The PSi waveguide was firstly silanized with APTES and then modified with SPDP, used for the immobilization of thiolate-ssDNA. The results highlighted a measured LoD of 3 nM when PNA/DNA hybridization occurred [104].

Recently, Moretta et al. explored the introduction of a specific PNA sequence onto a hybrid GO-PSi platform in order to develop an assay for the rapid detection of Brugada Syndrome (BS). To obtain the device, freshly etched PSi was passivated via thermal hydrosilylation with undecylenic acid, followed by the PEGylation process via EDC/NHS. By using the same covalent approach, first graphene oxide (GO) and finally the PNA were immobilized on the surface of the porous matrix. The PNA probe, used in this study, was properly designed in order to detect a punctual mutation related to the SCN5A gene, responsible for BS. The hybridization between the PNA-GO-PSi device and the DNA target was quantified by using reflectivity analysis and fluorescence microscopy, showing an LoD of 25 ± 2 µM and 18 ± 3 µM, respectively [37].

#### 3.3.2. Porous Silicon Aptasensors

Although the antibodies were extensively used as detection probes in various types of biosensors over the last 70 years, they suffer from several drawbacks (i.e., high cost, large size, and instability), making the use of alternative probes essential.

In this regard, several scientific papers published in the last 20 years, have highlighted the possibility of replacing the antibodies with aptamers, as promising capture probes for biosensing applications. The idea of using aptamers was initially proposed in 1990 [123]. Aptamers are short (usually from 20 to 60 nucleotides) single-strand DNA or RNA oligonucleotides, with a specific 3-dimensional structure that ensures the binding to a specific target. They are commonly synthetized by using the SELEX (Systematic Evolution of Ligands by Exponential enrichment) technique, which consists of repeated rounds of an in vitro selection and amplification process. This methodology allows not only the engineering of these biomolecules toward different targets (i.e., small molecules, proteins, whole cells) but also the reproducibility of the synthetic process [124,125]. Aptamers are considered as strong rivals to antibodies, showing a high affinity towards their targets thanks to their dissociation constants (K_d_), ranging from picomolar to nanomolar. Therefore, their capability to discriminate any structural differences among analogue targets makes them highly selective [126]. The aforementioned properties, coupled with cost-effectiveness, simple synthetic process, stability in non-physiological conditions as well as resistance to degradation and denaturation, suggest the use of the aptamers as an interesting alternative to antibodies [127,128].

The versatility of aptamers as recognition elements makes them extensively explored in the construction of aptasensors [21]. Segal is a pioneer in the use of aptamers in PSi optical devices, widely used for the detection of several proteins or whole-cells, as demonstrated in several published papers [79,105,129,130,131]. An example was provided by the use of the aptamer Hemag1P, a 78-nucleotide sequence, employed as a capture probe for the real-time monitoring of *Lactobacillus acidophilus,* a Gram-positive bacterium, present in fermented and dairy-containing food products. A PSi Fabry-Pérot thin film, used as a substrate, was oxidized, silanized with MPTMS, and finally incubated with the aptamer that specifically targets S–proteins, on the outer membrane of living bacteria. The large size of *L. acidophilus* cells prevented their infiltration into the porous matrix, leading to the direct capture of bacteria onto the PSi platform. Therefore, the monitoring of aptamer–cell interaction was performed by measuring the changes in the intensity of the reflectivity spectrum, caused by the light scattering of bacteria residing on the biosensor [132]. The aforementioned aptasensor demonstrated its ability to detect relatively low concentrations of *L. acidophilus* cells (low as 10^6^ cells per mL), discriminating between live and dead bacteria. The development of such a biosensor could be useful to monitor probiotic amount in functional food and pharmaceuticals.

Unfortunately, the insufficient sensitivity of label-free PSi biosensors, due to the hindered diffusion of analyte into the porous matrix, represents a limiting factor. In this context, Arshavsky–Graham et al. reported a proof-of-concept work in which the specific affinity of an aptasensor to its target was paired to the abovementioned ITP technique. The device was integrated into a microfluidic system, and prior to integration with PDMS microchannels, the oxidized PSi was modified with APTES. Finally, the EDC solution and the aptamer were pumped under vacuum into the microchannels to guarantee a covalent bond of the probe to the device. The suggested assay, also applicable in complex media, allowed to enhance the biosensor’s sensitivity up to 1000-fold, measuring an LoD of 7.5 nM, when the interaction between the aptamer and the His-tagged protein used as model, occurs [79].

**Figure 7:**
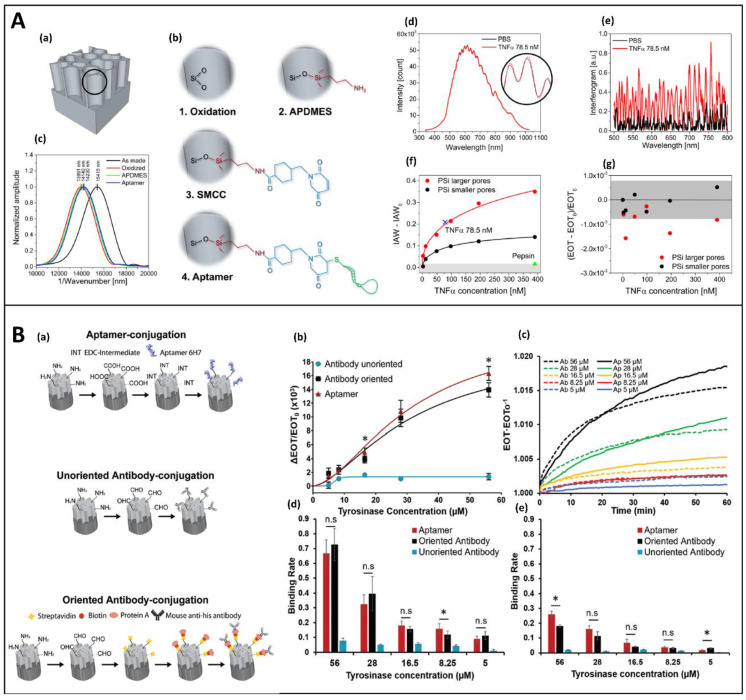
(**A**) (a) Cartoon of the as-prepared PSi platform. (b) Four-step protocol used to immobilize the aptamer probe into the PSi structure consisting of thermal oxidation, silanization with APDMES, grafting of SMCC, and aptamer binding; (c) FFT spectra measured after each functionalization steps; (d) reflection spectra acquired before (black line) and after injection of TNFα (red line) with (e) corresponding spectral interferogram; (f) IAW signals obtained in the range of TNFα tested (3–390 nM) for PSi with smaller (black line) and larger (red line) pores. The blank IAW_0_ value is subtracted from the data; (g) calibration curve over the whole range of the tested TNFα obtaining using the EOT value as output signal. Reprinted with permission from Reference [77]; Copyright (2016) American Chemical Society. (**B**) (a) Scheme of the immobilization process of the 6H7 aptamer (upper panel) and antibodies, via random and oriented conjugation (lower panel) into PSiO_2_; (b) EOT changes of the three type biosensors after the exposure to different concentration of his-tagged-tyrosinase; (c) EOT changes vs time of oriented antibody and aptamer onto PSiO2 upon exposure to different concentrations of his-tagged-tyrosinase; (d,e) binding rates at 10 min and 60 min obtained after the incubation of the biosensors with dif-ferent concentrations of tyrosinase. (ns) or (*) indicate the non-significant or statistically significant difference, respec-tively, between the total binding or the binding rate of the oriented antibody and aptamer-based biosensors. Reprinted with permission from Reference [133]; Copyright (2020) The Royal Society of Chemistry 2020.

The sensitivity and the LoD of PSi aptasensors were improved by Barillaro et al. with a new post-processing analysis of the reflectance signal, instead of the conventional FFT reflectance (Figure 7A). This methodology, known as interferogram average over wavelength (IAW) reflectance spectroscopy, consisted of the calculation of averaged interferometric values over the spectral range of interest. This methodology was applied for the detection of tumor necrosis factor alfa (TNFα) at a concentration down to 3.0 nM. Moreover, Mariani et al. showed that the diffusion of biomolecules into the pores could be improved and the protein detection was enhanced by a factor 10^4^ with respect to nonamplified label-free PSi biosensors, by combining a novel PSi structure (with a pore size of ~80 nm) with the IAW technique. The following device was obtained by immobilizing a thiol modifier-aptamer on APDMES-modified PSiO_2_ through SMCC [77].

A further example of a PSi-based biosensor was proposed for the detection of human α-thrombin. Human α-thrombin, also known as coagulation factor II, is a serine protease that converts soluble fibrinogen into insoluble strands of fibrin, promoting blood clotting. High levels of thrombin in the blood could be a cause of several pathological coagulation diseases. For this reason, devices that are able to detect the thrombin levels in the blood with high selectivity, low LoD, and in a short time are urgently required. In this context, Terracciano et al. successfully demonstrated the development of a new label-free optical aptasensor, realized by in situ synthesis of a 15-mer thrombin binding aptamer (TBA_TT_), covalently conjugated to the APTES-modified macroporous silica surface. The in situ synthesis shows many advantages—an increased density of the bioprobe on the surface, enhancement of the device sensitivity, automatable fabrication process, and possibility to functionalize the surface in microarrays. Moreover, the PSi surface functionalization with TBA (F_TBA_) was estimated to be (1.92 ± 0.03) 10^−5^ mol/g, considering a PSi sample having a weight of 0.2 mg. The ratio F_TBA_/SSA (specific surface area, which was 199 m^2^ g^−1^ for as-etched PSi) was used to calculate the PSi functionalization in terms of nmol cm^−1^, resulting in 0.0125 ± 0.0002 nmol cm^−2^. The LoD reached by the aptasensor was 1.5 ± 0.3 nM. Moreover, the high selectivity and regenerability of the biosensor were proved. [106].

A recent interesting study, conducted by Arshavsky-Graham et al., compared, for the first time, the performances of an aptamer, a randomly immobilized antibody and an oriented antibody on the PSi surface for the his-tagged-tyrosinase detection, as reported in Figure 7B. A PSiO_2_ substrate, modified with APTES and succinic acid, was used to covalently link the aptamer to the surface via EDC/NHS chemistry. Silanized PSIO_2_ was functionalized with GA for the immobilization of antibodies. The direct exposure of antibodies to GA resulted in a random orientation. Vice versa, a modification of GA-silanized-PSiO_2_ with streptavidin (SA) and biotinylated-PrA allowed the right orientation of antibodies.

In brief, while unoriented antibodies on the PSi surface caused a poor biosensing performance, aptamer, and oriented antibody-based biosensors had similar results in terms of LoD, binding rate, and selectivity. Nevertheless, the superiority of the aptasensors was represented by the faster and cost-effective fabrication, as well as the possibility to store the device in dry conditions. Furthermore, PSi aptasensors could be regenerated thanks to the reversible denaturation process of aptamers [130,133]. Performances of the device could be improved through a lower surface coverage. Indeed, a reduced density surface allows minimizing the effects of steric hindrance and the electrostatic repulsion of the negatively-charged DNA molecules at higher concentrations [105,130,134].

## 4. PSi Biosensors for Analytes Detection in Complex Matrices

Many studies highlight the ability of biosensors to discriminate between target and non-target sequences, reaching a high sensitivity level. Although most of these analyses were carried out in clean buffers, the detection of analytes in complex media is still a challenge. The cross-reactivity and the non-specific attachment of biomolecules must be avoided in order to obtain a reliable result. This issue could be achieved by treating the surfaces with blocking agents (i.e., Tris, BSA, PEG). In some cases, additional steps, including pre-treatment or sample separation through suitable methodologies, are required to improve the specificity. Involving PSi as a transducer material for the detection of analytes in complex matrices, not only requires a proper surface stabilization, but also the appropriate tailoring of the porous matrix. The most common real samples are blood or serum, saliva, water, bacterial lysates, and human isles of Langerhans [79,134,135,136].

Bonanno et al. developed a PSi Bragg-mirror-competitive test to detect opioids in urine specimens. They demonstrated that, by varying the device chemical surface and volume of urine sample added to the porous matrix, sensitivity and specificity of the assay could be improved. Additionally, the fabrication of PSi layers, with different porosity, improved the infiltration of macromolecules through the pores. Good results were obtained by using a device that was previously oxidized, amino-silanized, and subsequently, exposed to bovine serum albumin (BSA). Finally, a morphine analogue (M3G) was covalently bound to lysine groups exposed on the BSA-PSi platform. The free drugs, contained in urine samples and analogues, bound to the matrix, competed for the binding site of the antibodies. An indirect methodology was used to detect the free drug, measuring an LoD of 0.018 µM and making the platform appealing for POC applications [137,138].

The foodborne diseases represent a public health problem, caused by the contamination of food with several types of microorganisms or mycotoxins that could occur during the several steps of food production. The ingestion of contaminated food is the cause of serious complications. For this reason, the rapid detection of bacterial contamination in the complex food industry water is crucial to prevent foodborne diseases. This issue was pursued by Massad-Ivanir et al. They designed a PSi-based biosensor for the real-time detection of *E. coli* in complex water samples. The silanized-PSi was modified with GA followed by exposure to SA. Finally, a biotinylated *E. coli* antibody was bound to the device. In the biosensing experiment, the interaction between *E. coli* cells-antibody was monitored as a decrease in the intensity of the reflected light, demonstrating an LoD of 10^3^ cells mL^−1^ in the food industry water, without pre-enrichment of the sample. Such a device could be considered a promising tool to detect contaminations during product processing, reducing the risk of the spread of pathogens [136].

A huge step forward was taken by the Voelcker group, which investigated the use of PSi optical rugate filter like an in vivo biosensor. First, the stability and the biocompatibility of thermally oxidized and thermal hydrocarbonized devices were compared in in vitro studies. Data obtained demonstrated that not only did the THC-PSi maintain its structural and optical integrity, but its pre-incubation in cell medium (DMEM) for 10 days made the material not cytotoxic. As a result, the PITHC-PSi, subcutaneously implanted in a murine model, revealed that the optical signal was recorded through the skin of the mice 1 week after implantation, emphasizing how the stabilization of the surface is important to minimize the degradation of the device in physiological media [45].

## 5. Conclusions

The well-established and low-cost fabrication processes, the versatility of the material, and its optical and physical properties, together with the high internal surface area, makes PSi an appealing material for the development of optical biosensors with applications in food safety, environmental monitoring, and medical diagnostics. To this aim, the knowledge of surface chemistry modification approaches plays a crucial role in PSi-based devices to protect the PSi from degradation and provide functional groups that are useful for the attachment of biorecognition probes. This aspect is extensively examined in this review, together with an accurate exploration of the recent advancements in optical PSi-based biosensors for the rapid detection of different analytes. The attention was mainly focused on identifying the different adopted strategies to improve the biosensing performances in terms of stability, sensitivity, and detection limit, starting from the bioprobe immobilization.

Although the detection of analytes in clean samples is widely validated, identifying a specific molecule in real samples still represents a challenge. Moreover, even though a few examples are already reported in the literature, further efforts are needed to enhance biosensor stability in complex sample matrices and minimize the non-specific adsorption of biomolecules. This issue could be achieved through a practical surface modification strategy.

Other studies are necessary to obtain the miniaturization of the devices that could become important for the development and commercialization of POC devices.

## Figures and Tables

**Table 1:** Summary of detection of various Biomolecules using PSi optical device as a transducing substrate.

PSi Structure	Transduction Mechanism	Type of Bioprobe	Capture Probe	Analyte	LoD	Ref.
TiO_2_ PSi	Reflectivity	Antibody	Anti-SLP	S-layer protein	0.70 ± 0.37 pM	[80]
Si nanopore array	Reflectivity	Antibody	*E. coli* antibody	*E. coli*	10^3^ to 10^7^ CFU mL^−1^	[84]
Fabry-Pérot	Reflectivity	Antibody	Anti-HSP70	HSP70	1290 ± 160 ng/mL	[83]
Single layer	Photoluminescence	Antibody	Anti-OTA	Ochratoxin A	4.4 pg mL^−1^	[92]
Si NWs	Photoluminescence	Antibody	Anti-CRPs	C-reactive protein	1.6 fM	[93]
PSi layer covered with thin layer of Au	Photoluminescence	Antibody	Anti-AFB1	Aflatoxin B1	2.5 ± 0.5 pg/mL	[94]
Bragg mirror	Photoluminescence	Antibody	Anti-p38	*Echinococcus granulosus*	300 fg mL^−1^	[35]
Single layer	Reflectivity	Enzyme	Horseradish peroxidase	Ag^+^, Pb^2+^ and Cu^2^	60–120 ppb	[97]
Single layer	Reflectivity	Enzyme	1-Naphthyl-N-acetyl-β-D-glucosaminide	NGAse substrate	0.51 µM/min	[98]
Microcavity	Fluorescence (FRET-based)	Enzyme	Fluorogenic peptides	Sortase A and MMPs	4.6 × 10^−8^ M	[39]
Microcavity	Fluorescence	Enzyme	Resazurin	L-lactate dehydrogenase	0.08 U/mL	[99]
SiNWs	Photoluminescence	Enzyme	Glucose oxidase	Glucose	1.06 µM	[40]
Single layer	Reflectivity	DNA	DNA		1 × 10^−9^ M	[100]
Double Bragg mirror	Reflectivity	DNA	Complementary and partially complementary DNA	AOB gene	27.1 nM and 35 nM	[71]
Microcavity	Photoluminescence	DNA	DNA	-		[101]
Bragg mirror	Digital fluorescence microscopy	DNA	DNA	*-*	88 pM	[102]
Au/PSi	Photoluminescence	DNA	DNA	*-*	328.7 nM	[103]
Ring resonator	Reflectivity	DNA	PNA	*-*	3 nM	[104]
Single layer	Reflectivity and Fluorescence	PNA	DNA	SCN5A gene	25 ± 2 µM and 18 ± 3 µM	[37]
Single layer	Reflectivity	Aptamer	Bacteria	*Lactobacillus acidophilus*	10^6^ cells mL^−1^	[105]
Single layer	Reflectivity	Aptamer	40-mer anti-HIs-tag 6H7 aptamer	His-tagged protein	7.5 nM	[79]
Single layer	Reflectivity and IAW	Aptamer	28-mer anti TNF *α aptamer*	*TNFα*	3.0 nM	[77]
Single layer	Reflectivity	Aptamer	17-mer thrombin binding aptamer	*Human α-thrombin*	1.5 ± 0.3 nM	[106]

## Data Availability

Not applicable.
